# Multitask optimization and convergence stability with hierarchical feature learning for self guided optimization

**DOI:** 10.1038/s41598-026-36622-y

**Published:** 2026-01-27

**Authors:** Khalid Mahmood, Maha M. Althobaiti, Mahmood Ul Hassan, Sonia Khan, Maryam Khan, Muaadh A. Alsoufi

**Affiliations:** 1 Engineering and Technical Specializations Unit, Applied College, King Khalid University, 61421 Muhayil, Aseer Kingdom of Saudi Arabia; 2 Department of Computer Science, College of Computing and Information Technology, Taif University, P.O.Box 11099, 21944 Taif, Saudi Arabia; 3 Department of Computer Science, IIC University of Technology, 121206 Phnom Penh, Cambodia; 4 Department of Youth Affairs, Youth Office Mansehra, Directorate of Youth Affairs, Government of Khyber Pakhtunkhwa, Haripur, 22620 Islamic Republic of Pakistan; 5 Department of Computer Science, Government Post Graduate College For Women, Haripur, Khyber Pakhtunkhwa Islamic Republic of Pakistan; 6 Faculty of Engineering and Information Technology, Taiz University, 6803 Taiz, Yemen

**Keywords:** Engineering, Mathematics and computing

## Abstract

The optimization process of multimodal multitask architectures faces three major problems which include unstable optimization and unresolved cross-task interference and insufficient alignment between different feature views. The solution of these system failure points needs direct management of view-specific relationships and task-dependent feature extraction and multi-instance data processing methods. The Unified Multitask and Multiview Deep Architecture (UMDA) solves all optimization problems through its four interconnected computational blocks which operate as a unified system. The Hybrid Cross-View Attention module generates two types of attention operators which establish controlled inter-view relationships through entropy-based concentration mechanisms and cross-view consistency penalties and dispersion constraints that stop modalities from collapsing into each other. The Adaptive Task-Specific Branching module uses dual-path factorization to identify common elements in task projections which generates influence matrices that handle hierarchical task relationships through penalty functions for divergence and consistency. The Graph-Based Multi-Instance Pooling operator processes multi-instance data by building graphs and performing Laplacian propagation and structural signature aggregation based on higher-order tensor interactions that follow entropy and graph-smoothness rules. The Self-Guided Learning method achieves stable optimization through two mechanisms which use gradient magnitudes to adjust task-specific learning rates and combine weighted gradients to reduce objective function variance. The combined mechanisms in the system achieve 88.3% multitask classification accuracy and 0.973 cross-view feature consistency and 4.2% gradient variance reduction during identical training and resource conditions.

## Introduction

Deep learning models use two essential methods to create generalized feature representations for multiple predictive targets, which are multitask learning (MTL) and multi-view representation learning (MVRL)^1–4^. The construction of shared parameter spaces in MTL enables models to find common features between tasks, while reducing overfitting and improving their performance on particular tasks^5–8^. The combination of different modalities through MVRL produces complete representations which become more robust due to the unique noise patterns found in individual data sources^9–12^. The combination of these two approaches enables developers to create sophisticated predictive systems that solve complex real-world problems that arise from multiple linked data streams^7,13–15^. The essential value of these technologies exists in medical imaging and bioinformatics and natural language processing and remote sensing applications because they need to learn from different types of data sources. The combination of imaging data with genomic information and demographic details enables clinical decision support systems to function properly. Autonomous navigation systems require visual data processing with spatial and temporal information integration to achieve effective operation. The development of these frameworks faces difficulties because they need to handle multiple view relationships while maintaining task uniqueness and operating at high speeds in complex multimodal data environments^12,16–18^.

### Challenges in current research

The existing MTL and MVRL frameworks contain essential weaknesses that prevent them from functioning properly in operational environments. The performance of multitask approaches deteriorates due to negative transfer that occurs when task gradients from unrelated tasks create destructive interference that leads to decreased performance in specific objectives^2,3,6,19^. The combination of task-specific and global features through fixed feature-sharing mechanisms results in the loss of specialized knowledge. Real-world datasets with various data distributions and multiple connected tasks increase the severity of these problems when models are applied to them. The combination of different views through basic methods that use concatenation or simple pooling produces duplicate information while missing complex connections between different data types. The current pooling methods fail to detect relationships between instances because they do not consider the relational structure that exists between them in a bag^7,10,19^. The optimization methods operate independently with set methods to handle task gradients, resulting in unstable training and unpredictable results on unseen data^2–4,8^.

### Motivation and research objectives

The current multitask and multiview learning frameworks have various limitations, which motivate researchers to develop innovative methods that combine task-dependent flexibility with cross-view relationship modeling and structural feature combination. The development of models for medical diagnostics and industrial defect detection and multimodal classification requires better prediction accuracy and stable operation, and efficient performance and interpretability. Current solutions lack mechanisms to control redundant information and maintain stable optimization, making them susceptible to performance degradation and biased feature extraction (li2024mosdnet)^6,7,11,20^. Research develops a unified framework that regulates both common and task-dependent representation strength through methodical integration of instance features and keeps optimization stable through task relationship control^2–4,15^. The proposed solution addresses three major problems in feature combination and task conflicts and gradient instability while providing stable convergence for different types of data^7,12,14,21^.

### Proposed technical approach

The proposed framework uses a hierarchical multitask and multiview learning system which includes graph-based, attention-driven, and gradient-adaptive components. The Hybrid Cross-View Attention mechanism identifies first-order and second-order view dependencies through entropy-based and diversity-based and consistency-based regularization to eliminate duplicate information while improving feature distinction^22^. The system includes Adaptive Task-Specific Branching, which divides common and exclusive task information, determines task relationships, and enhances feature transmission through divergence and consistency penalties. The Graph-Based Multi-Instance Pooling module creates similarity graphs between instances before using Laplacian propagation to combine features through second-order tensor operations^19^. The Self-Guided Learning method achieves convergence stability through task-specific optimization control, which adjusts learning rates and gradient weights based on similarity-based dependencies. The major contributions are summarized as follows:

- The two-level attention module improves view dependencies at first and second order through entropy and diversity and consistency regularization, which eliminate unimportant information to preserve discriminative features.
- The dynamic pooling method creates instance similarity graphs to perform Laplacian-based propagation and second-order tensor aggregation for detecting structural patterns which standard pooling methods fail to detect.
- The gradient-aware learning system adjusts task-specific learning rates based on gradients while using similarity reweighting to match gradients and implements convergence stabilization through variance penalty on inter-task differences under a combined multi-objective loss function.

### Structure of the article

The structure of the article begins with Sect. 2, which conducts an essential evaluation of current research findings. The UMDA framework receives its definition through Sect. 3 that explains its components and presents a new optimization solution. Section 4 shows the results of the system experimental tests. The research findings are evaluated in Sect. 6, which also presents directions for upcoming studies. The article concludes in Sect. 7.

## Related work

The research area of multi-task and multi-view learning has gained increasing popularity because these methods combine multiple input sources to discover vital data patterns which achieve best results for multiple related tasks. Research studies have developed dynamic feature interaction frameworks for vision-based perception through structured fusion methods that optimize information exchange between tasks^23^. The combination of cross-view feature fusion with spatio-temporal cues in multi-target detection systems results in better object localization performance and stronger generalization abilities^24^. The combination of static and dynamic neighbors in multi-task recommendation systems produces better results by embedding refinement control^25^. The combination of multiple data types through structured characteristic alignment produces better tracking results and improved classification accuracy according to^26,27^. The current methods fail to produce acceptable results in complex multi-instance environments because they do not properly manage instance relationships and task-related constraints. The proposed solution implements a dynamic task-based optimization system that performs structured feature fusion to address existing problems, as shown in Table 1.

**Table 1:** Comparison of identified limitations in existing approaches and their resolution in the proposed framework.

Ref.	Identified limitation	Proposed resolution
^23^	Fixed feature interactions reduce adaptability in multi-task learning.	Introduces hybrid cross-view attention mechanism (HCVA) to dynamically refine shared and task-specific features.
^24^	Cross-view fusion lacks structured feature alignment for detection tasks.	Implements a graph-based feature integration strategy to preserve structural dependencies.
^25^	Static neighbor modeling in recommendation leads to performance degradation.	Develops adaptive task-specific branches that refine contextual embedding updates.
^26,27^	Multi-modal fusion lacks flexibility in tracking applications.	Uses a hybrid graph representation to maintain inter-instance dependencies.
^28^	Multi-instance knowledge distillation struggles with inconsistent task scaling.	Adopts self-optimizing gradient regulation to balance task-specific learning objectives.
^29^	EEG-based feature fusion lacks hierarchical integration strategies.	Incorporates hierarchical graph-based multi-instance pooling for improved aggregation.
^30^	Multi-view datasets lack structured feature fusion for real-world adaptability.	Proposes a dynamic task-aware optimization scheme to ensure robust multi-task performance.
^31^	Fault diagnosis systems rely on static multi-task processing.	Introduces an adaptive multi-task fusion model with context-aware optimization.
^32^	Stereoscopic image discomfort prediction suffers from suboptimal feature representation.	Implements attention-driven hierarchical fusion to refine visual discomfort estimation.
^33^	Robotic manipulation lacks efficient historical learning integration.	Utilizes multi-view hierarchical learning for structured feature aggregation.
^34^	Sentiment analysis struggles with imbalanced multi-task weighting.	Develops self-guided task weighting to improve sentiment classification consistency.
^35^	Multi-view feature fusion in medical diagnosis lacks interpretability.	Employs structured graph-based pooling to enhance diagnostic reliability.

Research has been conducted to study knowledge distillation methods in multi-task, multi-view systems for myocardial infarction detection through the analysis of multi-instance aggregation methods that improve the extraction of cardiovascular characteristic^28^. The field of emotion recognition research uses multi-task latent feature fusion to combine deep electrophysiological signal features for improved classification performance^29^. The development of assistive driving perception requires structured multi-view and multi-modal and multi-task datasets that require adaptive learning systems to handle different sensor types^30^. Conventional methods using static pooling fail to detect complex feature relationships because their methods do not understand the precise connections between individual samples^31^. The application of multi-view representation learning in stereoscopic image discomfort prediction faces difficulties because of the significant differences between learned embeddings. The combination of historical learning with the fusion of structured features through hierarchical attention mechanisms produces superior results in robotic manipulation according to^33^. The optimization of feature interactions in multimodal sentiment analysis needs dynamic multi-tasking and contrastive learning strategies which prove that structured optimization methods are essential. Deep multi-view fusion techniques have been applied in breast cancer classification to improve diagnostic precision through multimodal integration^35^.

## Proposed framework: UMDA

**Fig. 1:**
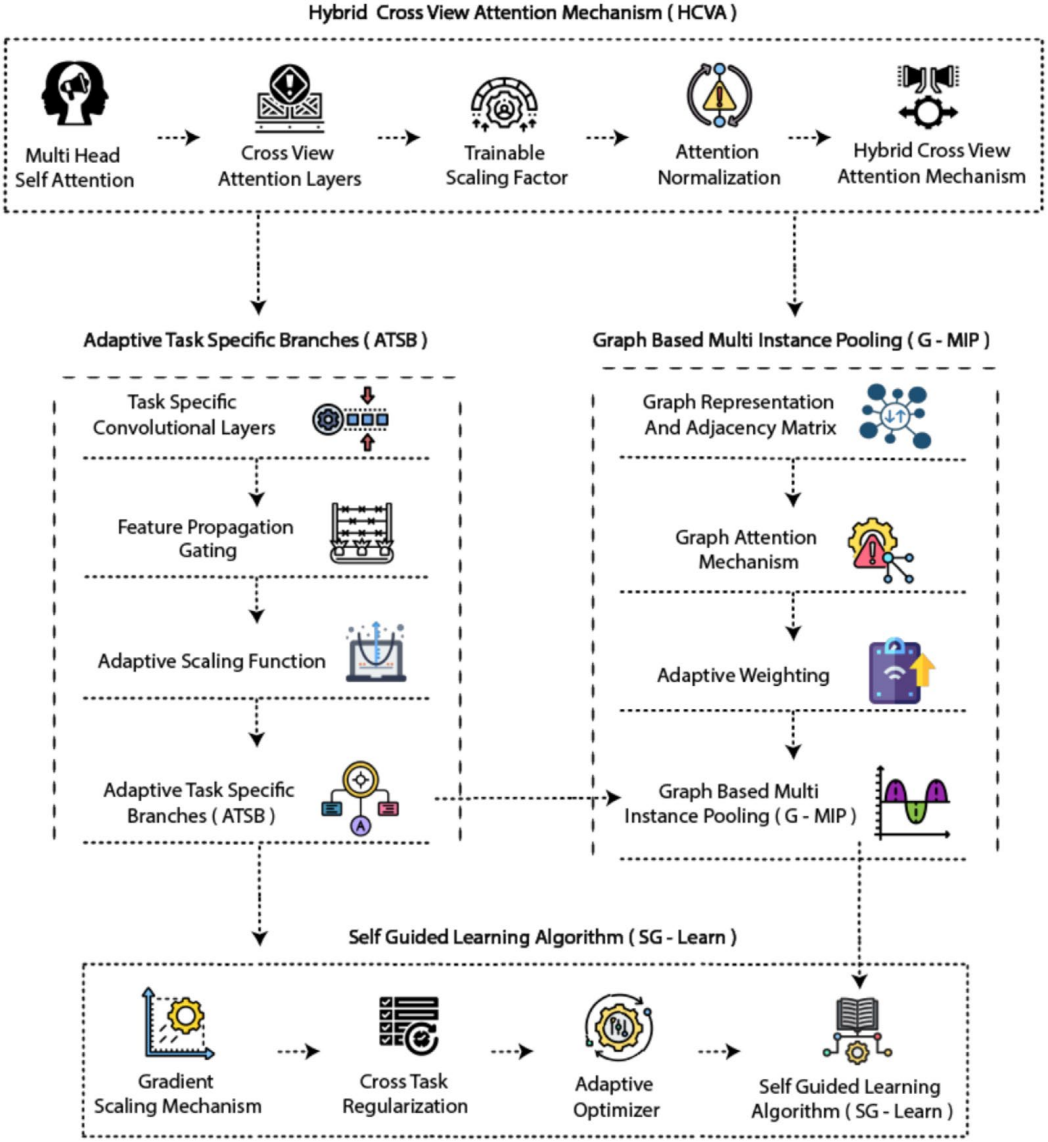
The UMDA framework unites hybrid cross-view attention (HCVA) for hierarchical feature extraction with adaptive task-specific branches (ATSB) for task-aware learning and graph-based multi-instance pooling (G-MIP) for structured feature aggregation and self-guided learning (SG-Learn) for dynamic optimization.

The proposed framework uses a systematic multi-task multi-view learning system that unites standard features with unique characteristics from each task. The optimization system controls feature combinations to reduce task conflicts while allowing users to customize their views. The workflow of the UMDA framework consists of components HCVA and ATSB and G-MIP and SG-Learn which work together as shown in Fig.  1.

### Hybrid cross-view attention mechanism (HCVA)

To establish a robust multi-view feature extraction mechanism, the hybrid cross-view attention mechanism (HCVA) is designed as a multi-order adaptive transformation framework (see Algorithm 1). The mechanism introduces structured self-attention operators that perform hierarchical aggregation of view-specific features while preserving both shared and task-conditioned representations.

**Algorithm 1:**
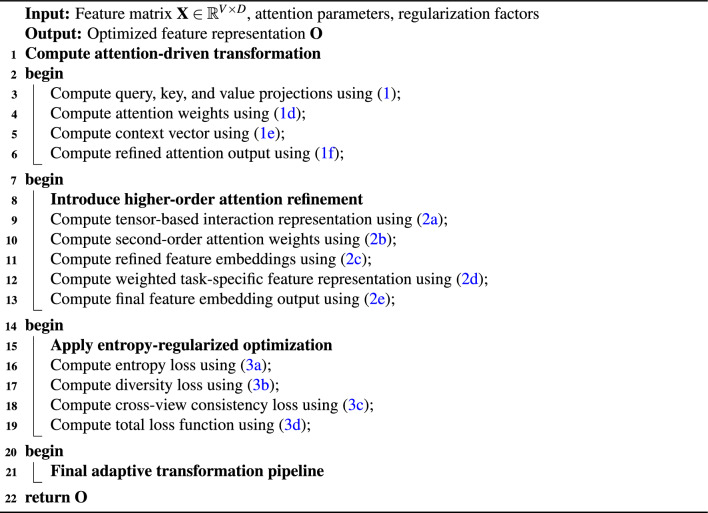
Hybrid cross-view attention mechanism (HCVA)

For an input feature matrix $\textbf{X} \in \mathbb {R}^{V \times D}$, where *V* denotes the number of views and *D* is the dimensionality of each view-specific feature vector, the first-order attention-driven transformation is defined as follows:

1a $$\begin{aligned} \textbf{Q}_v= & \textbf{W}_q \textbf{X}_v, \end{aligned}$$

1b $$\begin{aligned} \textbf{K}_v= & \textbf{W}_k \textbf{X}_v, \end{aligned}$$

1c $$\begin{aligned} \textbf{V}_v= & \textbf{W}_v \textbf{X}_v, \end{aligned}$$

1d $$\begin{aligned} \textbf{A}_{uv}= & \frac{\exp (\textbf{Q}_u \textbf{K}_v^\top )}{\sum _{j} \exp (\textbf{Q}_u \textbf{K}_j^\top )}, \end{aligned}$$

1e $$\begin{aligned} \textbf{Z}_u= & \sum _{v} \textbf{A}_{uv} \textbf{V}_v + \alpha \cdot \textbf{X}_u, \end{aligned}$$

1f $$\begin{aligned} \textbf{H}_u= & \sigma (\textbf{Z}_u) + \phi (\textbf{X}_u) + \epsilon \cdot \textbf{X}_u. \end{aligned}$$

In (1a), $\textbf{Q}_v \in \mathbb {R}^{d_q}$ is the query representation of the *v*-th view, obtained by a linear projection matrix $\textbf{W}_q \in \mathbb {R}^{d_q \times D}$ applied to the view-specific feature vector $\textbf{X}_v \in \mathbb {R}^{D}$. In (1b), $\textbf{K}_v \in \mathbb {R}^{d_k}$ denotes the corresponding key representation, computed through a projection matrix $\textbf{W}_k \in \mathbb {R}^{d_k \times D}$. Equation (1c) defines the value representation $\textbf{V}_v \in \mathbb {R}^{d_v}$ with projection matrix $\textbf{W}_v \in \mathbb {R}^{d_v \times D}$. The indices *u* and *v* enumerate the source and target views, respectively. In (1d), $\textbf{A}_{uv} \in \mathbb {R}$ is the normalized attention weight that quantifies the importance of view *v* when updating view *u*, where the numerator uses an exponential of the inner product between $\textbf{Q}_u$ and $\textbf{K}_v$, and the denominator normalizes over all views indexed by *j*. Equation (1e) constructs the context vector $\textbf{Z}_u \in \mathbb {R}^{d_v}$ as a weighted aggregation of value vectors $\textbf{V}_v$ across views, with an additional residual contribution $\alpha \cdot \textbf{X}_u$, where $\alpha \in \mathbb {R}$ is a learnable scalar scaling factor and $\textbf{X}_u$ is the original feature of view *u*. In (1f), $\textbf{H}_u \in \mathbb {R}^{d_h}$ represents the first-order refined representation for view *u*, obtained by applying a nonlinear activation function $\sigma (\cdot )$ to $\textbf{Z}_u$, adding a nonlinear residual mapping $\phi (\textbf{X}_u)$, and incorporating another residual term $\epsilon \cdot \textbf{X}_u$ with scalar coefficient $\epsilon \in \mathbb {R}$. The functions $\sigma (\cdot )$ and $\phi (\cdot )$ are differentiable nonlinear operators, and $d_h$ denotes the dimensionality of the refined feature space. To extend beyond first-order attention and explicitly encode higher-order dependencies, HCVA introduces a second-order refinement stage based on tensor interactions over the intermediate representations:

2a $$\begin{aligned} \textbf{T}_{uvw}= & \textbf{Z}_u \otimes \textbf{Z}_v \otimes \textbf{Z}_w, \end{aligned}$$

2b $$\begin{aligned} \textbf{B}_{uv}= & \frac{\sum _{w} \textbf{T}_{uvw}}{\sum _{j} \sum _{w} \textbf{T}_{ujw}}, \end{aligned}$$

2c $$\begin{aligned} \textbf{F}_u= & \sum _{v} \textbf{B}_{uv} \textbf{H}_v + \lambda \cdot \textbf{H}_u, \end{aligned}$$

2d $$\begin{aligned} \textbf{Y}_u= & \gamma \textbf{F}_u + (1 - \gamma ) \textbf{H}_u, \end{aligned}$$

2e $$\begin{aligned} \textbf{O}_u= & \delta (\textbf{Y}_u) + \mu \cdot \textbf{X}_u. \end{aligned}$$

In (2a), $\textbf{T}_{uvw}$ is a third-order tensor that encodes joint interactions among the intermediate context representations $\textbf{Z}_u$, $\textbf{Z}_v$, and $\textbf{Z}_w$ associated with views indexed by *u*, *v*, and *w*. The operator $\otimes$ denotes the outer product, which produces a multi-way interaction structure capturing nonlinear correlations across three views simultaneously. Equation (2b) defines the second-order attention coefficient $\textbf{B}_{uv} \in \mathbb {R}$ by summing over the index *w* in the numerator and normalizing by the total tensor mass over all pairs (*j*, *w*) in the denominator. This normalization constrains $\textbf{B}_{uv}$ to act as a probability-like weighting function across views and stabilizes higher-order interactions. In (2c), $\textbf{F}_u \in \mathbb {R}^{d_h}$ is a refined representation of view *u* that aggregates the first-order outputs $\textbf{H}_v$ using second-order weights $\textbf{B}_{uv}$, with an additional residual term $\lambda \cdot \textbf{H}_u$, where $\lambda \in \mathbb {R}$ is a learnable scalar that controls the contribution of the self-view representation. Equation (2d) forms $\textbf{Y}_u \in \mathbb {R}^{d_h}$ by interpolating between $\textbf{F}_u$ and $\textbf{H}_u$ using a mixing coefficient $\gamma \in [0,1]$, which regulates the strength of second-order refinement relative to first-order attention. Finally, in (2e), $\textbf{O}_u \in \mathbb {R}^{d_o}$ denotes the final view-specific embedding, obtained by applying a nonlinear transformation $\delta (\cdot )$ to $\textbf{Y}_u$ and adding a residual connection from the original feature $\textbf{X}_u$ scaled by a learnable parameter $\mu \in \mathbb {R}$, where $d_o$ is the dimensionality of the final embedding space and $\delta (\cdot )$ is a differentiable activation or projection function. To regulate the attention process and improve optimization stability, HCVA incorporates an entropy-regularized loss formulation that constrains the attention maps at both first and second orders and enforces structured diversity across views:

3a $$\begin{aligned} \mathscr {L}_{\text {entropy}}= & -\sum _{u} \sum _{v} \textbf{A}_{uv} \log \textbf{A}_{uv} + \beta \sum _{u} \sum _{v} \textbf{B}_{uv} \log \textbf{B}_{uv}, \end{aligned}$$

3b $$\begin{aligned} \mathscr {L}_{\text {diversity}}= & \sum _{u} \left\| \textbf{Y}_u - \frac{1}{V} \sum _{v} \textbf{Y}_v \right\| _2^2, \end{aligned}$$

3c $$\begin{aligned} \mathscr {L}_{\text {cross-view}}= & \sum _{u} \sum _{v} \Vert \textbf{Y}_u - \textbf{Y}_v\Vert _2^2, \end{aligned}$$

3d $$\begin{aligned} \mathscr {L}_{\text {total}}= & \mathscr {L}_{\text {task}} + \lambda _1 \mathscr {L}_{\text {entropy}} + \lambda _2 \mathscr {L}_{\text {diversity}} + \lambda _3 \mathscr {L}_{\text {cross-view}}. \end{aligned}$$

In (3a), $\mathscr {L}_{\text {entropy}}$ penalizes low-entropy attention distributions to prevent collapse onto a single dominant view. The first summation term involves $\textbf{A}_{uv}$, the first-order attention weight between source view *u* and target view *v*, and applies the negative Shannon entropy across all view pairs. The second summation term acts on the second-order attention weights $\textbf{B}_{uv}$, scaled by a coefficient $\beta \in \mathbb {R}_{\ge 0}$, which tunes the relative strength of the second-order entropy contribution. Equation (3b) defines the diversity term $\mathscr {L}_{\text {diversity}}$, where $\textbf{Y}_u$ is the refined embedding of view *u*, and $\frac{1}{V} \sum _{v} \textbf{Y}_v$ is the mean embedding across all views. The squared $\ell _2$-norm encourages each view representation to deviate from the global mean, promoting representation diversity under a controlled quadratic penalty. In (3c), $\mathscr {L}_{\text {cross-view}}$ aggregates pairwise squared Euclidean distances $\Vert \textbf{Y}_u - \textbf{Y}_v\Vert _2^2$ between all view pairs (*u*, *v*), which enforces cross-view alignment by penalizing large discrepancies among refined embeddings. Equation (3d) defines the overall HCVA loss $\mathscr {L}_{\text {total}}$, combining a task-specific objective $\mathscr {L}_{\text {task}}$ with the three regularization components weighted by non-negative coefficients $\lambda _1$, $\lambda _2$, and $\lambda _3$. The term $\mathscr {L}_{\text {task}}$ encodes the supervised objective associated with the downstream tasks (for example, classification or regression), and the hyperparameters $\lambda _1$, $\lambda _2$, and $\lambda _3$ control the relative influence of entropy regularization, diversity enforcement, and cross-view consistency, respectively.

### Adaptive task-specific branches (ATSB)

The ATSB mechanism introduces a controlled task-adaptive transformation process in which shared and task-specific components are decoupled, refined, and subsequently recombined under regulated inter-task dependencies (Algorithm 2). This arrangement isolates task-conditioned variations while maintaining stable shared feature propagation.

**Algorithm 2:**
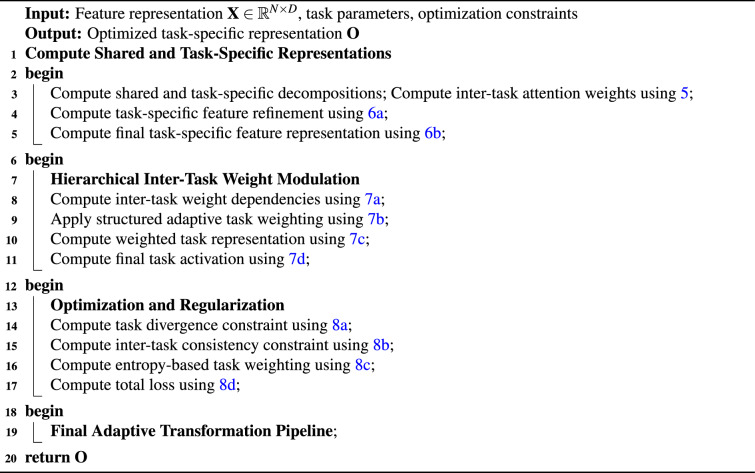
Adaptive task-specific branches (ATSB)

For an input matrix $\textbf{X} \in \mathbb {R}^{N \times D}$, the shared and task-specific partitions are computed as:

4a $$\begin{aligned} \textbf{X}_s= & \textbf{W}_s \textbf{X}, \end{aligned}$$

4b $$\begin{aligned} \textbf{X}_t= & \textbf{W}_t \textbf{X}. \end{aligned}$$

Equation 4a applies the shared projection matrix $\textbf{W}_s$ to extract global representational components, whereas Eq. 4b applies the task-specific projection matrix $\textbf{W}_t$ to isolate task-conditioned variations. Both mappings preserve dimensional consistency and establish the foundation for affinity-based refinement. The inter-task affinity matrix is computed as:

5 $$\begin{aligned} \textbf{A}_{st} = \frac{\exp (\textbf{X}_s^{\top } \textbf{X}_t)}{\sum _{j} \exp (\textbf{X}_s^{\top } \textbf{X}_j)}. \end{aligned}$$

Here, $\textbf{A}_{st}$ quantifies the directional influence of the shared representation $\textbf{X}_s$ on each task-specific component $\textbf{X}_t$, normalized across all tasks to prevent unbounded amplification. Task-specific refinement is then formulated as:

6a $$\begin{aligned} \textbf{F}_t= & \sum _{s} \textbf{A}_{st} \textbf{X}_s + \beta \textbf{X}_t, \end{aligned}$$

6b $$\begin{aligned} \textbf{H}_t= & \sigma (\textbf{F}_t) + \gamma \textbf{X}_t. \end{aligned}$$

Equation 6a blends attention-regulated shared components with the baseline task-specific input under the scaling coefficient $\beta$. Equation 6b applies a nonlinear activation $\sigma (\cdot )$ and an additive residual term $\gamma \textbf{X}_t$, stabilizing gradient propagation during refinement. The second refinement stage constructs structured inter-task dependencies:

7a $$\begin{aligned} \textbf{M}_{ts}= & \frac{\sum _{d} \textbf{H}_t^{d}\textbf{H}_s^{d}}{\sum _{j}\sum _{d} \textbf{H}_j^{d}\textbf{H}_s^{d}}, \end{aligned}$$

7b $$\begin{aligned} \textbf{G}_t= & \sum _{s} \textbf{M}_{ts} \textbf{H}_s + \lambda \textbf{H}_t, \end{aligned}$$

7c $$\begin{aligned} \textbf{Y}_t= & \omega \textbf{G}_t + (1-\omega )\textbf{H}_t, \end{aligned}$$

7d $$\begin{aligned} \textbf{O}_t= & \delta (\textbf{Y}_t) + \mu \textbf{X}_t. \end{aligned}$$

Equation 7a computes a normalized correlation-driven inter-task weighting matrix using elementwise interactions across feature dimensions *d*. Equation 7b aggregates task-conditioned responses under the modulation parameter $\lambda$. Equation 7c interpolates between the aggregated and original refined outputs using the mixing coefficient $\omega$. Equation 7d applies a final activation $\delta (\cdot )$ and reintroduces the raw task input $\mu \textbf{X}_t$ to preserve stability. To regulate interference across tasks, the following set of losses is introduced:

8a $$\begin{aligned} \mathscr {L}_{\text {divergence}}= & \sum _{t}\left\| \textbf{Y}_{t} - \frac{1}{T}\sum _{s}\textbf{Y}_{s} \right\| ^{2}, \end{aligned}$$

8b $$\begin{aligned} \mathscr {L}_{\text {consistency}}= & \sum _{t}\sum _{s}\Vert \textbf{Y}_{t} - \textbf{Y}_{s}\Vert _{2}^{2}, \end{aligned}$$

8c $$\begin{aligned} \mathscr {L}_{\text {entropy}}= & -\sum _{t}\sum _{s} \textbf{M}_{ts}\log \textbf{M}_{ts}, \end{aligned}$$

8d $$\begin{aligned} \mathscr {L}_{\text {total}}= & \mathscr {L}_{\text {task}} + \lambda _{1}\mathscr {L}_{\text {divergence}} + \lambda _{2}\mathscr {L}_{\text {consistency}} + \lambda _{3}\mathscr {L}_{\text {entropy}}. \end{aligned}$$

Equation 8a enforces dispersion between task outputs by penalizing deviation from their mean. Equation 8b penalizes large inter-task discrepancies, reinforcing coherence across task branches. Equation 8c constrains the weighting matrix $\textbf{M}_{ts}$ to avoid degenerate allocations. Equation 8d forms the composite objective incorporating the task-driven loss $\mathscr {L}_{\text {task}}$ and three regularization terms.

### Graph-based multi-instance pooling (G-MIP)

The G-MIP module constructs a structured relational model for multi-instance inputs by forming weighted graphs, applying spectral propagation, and integrating both first-order and higher-order structural interactions (Algorithm 3).

**Algorithm 3:**
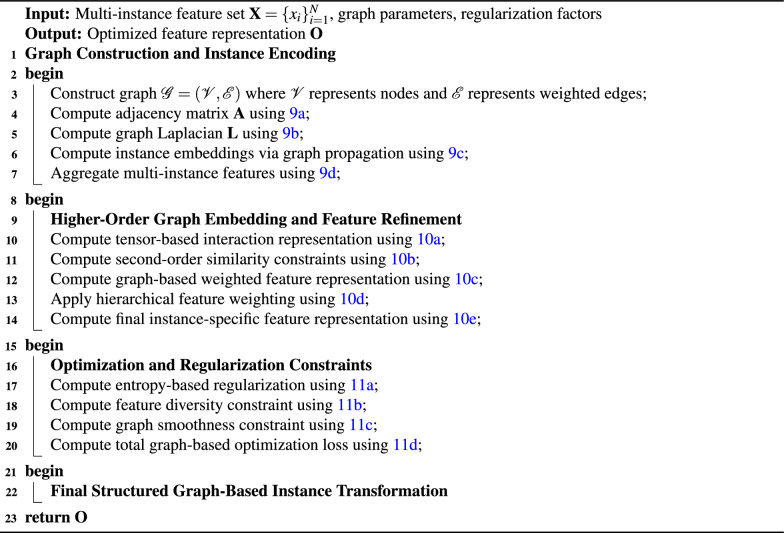
Graph-based multi-instance pooling (G-MIP)

For a multi-instance set $\textbf{X} = \{x_i\}_{i=1}^{N}$ with $x_i \in \mathbb {R}^D$, a weighted graph $\mathscr {G} = (\mathscr {V}, \mathscr {E})$ is formed. The connectivity structure is encoded through the adjacency matrix, the degree matrix, and the corresponding combinatorial Laplacian.

9a $$\begin{aligned} \textbf{A}_{ij}= & \exp \left( -\frac{\Vert \textbf{x}_i - \textbf{x}_j\Vert ^2}{\sigma ^2}\right) , \end{aligned}$$

9b $$\begin{aligned} \textbf{D}_{ii}= & \sum _{j}\textbf{A}_{ij}, \quad \textbf{L} = \textbf{D} - \textbf{A}, \end{aligned}$$

9c $$\begin{aligned} \textbf{H}^{(l+1)}= & \sigma \!\left( \textbf{L}\textbf{H}^{(l)}\textbf{W}^{(l)}\right) , \end{aligned}$$

9d $$\begin{aligned} \textbf{Z}= & \sum _{i=1}^N \alpha _i \textbf{H}_i, \qquad \alpha _i = \frac{\exp (\textbf{q}^\top \textbf{H}_i)}{\sum _{j=1}^N \exp (\textbf{q}^\top \textbf{H}_j)}. \end{aligned}$$

Equation 9a assigns an edge weight $\textbf{A}_{ij}$ between instances *i* and *j* by evaluating their Euclidean distance $\Vert \textbf{x}_i - \textbf{x}_j\Vert _2$ under a Gaussian kernel scaled by $\sigma >0$. Equation 9b defines $\textbf{D}$ as a diagonal degree matrix and constructs the Laplacian $\textbf{L}$, which governs spectral propagation. In Eq.  9c, $\textbf{H}^{(l)}$ denotes instance embeddings at layer *l*, $\textbf{W}^{(l)}$ is a learnable weight matrix, and $\sigma (\cdot )$ is a nonlinear activation. Equation 9d produces the aggregated representation $\textbf{Z}$ through an attention vector $\alpha _i$ parameterized by a query vector $\textbf{q}$. A tensor-based refinement stage then encodes higher-order structural dependencies.

10a $$\begin{aligned} \textbf{B}_{ijk}= & \textbf{H}_i \otimes \textbf{H}_j \otimes \textbf{H}_k, \end{aligned}$$

10b $$\begin{aligned} \textbf{S}_{ij}= & \frac{\sum _{k}\textbf{B}_{ijk}}{\sum _{m}\sum _{k}\textbf{B}_{imk}}, \end{aligned}$$

10c $$\begin{aligned} \textbf{G}_i= & \sum _{j}\textbf{S}_{ij}\textbf{H}_j + \lambda \textbf{H}_i, \end{aligned}$$

10d $$\begin{aligned} \textbf{Y}_i= & \gamma \textbf{G}_i + (1-\gamma )\textbf{H}_i, \end{aligned}$$

10e $$\begin{aligned} \textbf{O}_i= & \delta (\textbf{Y}_i) + \mu \textbf{H}_i. \end{aligned}$$

The third-order tensor $\textbf{B}_{ijk}$ in Equation 10a shows how embeddings $\textbf{H}_i$, $\textbf{H}_j$ and $\textbf{H}_k$ relate to each other. The similarity score $\textbf{S}_{ij}$ in Eq.  10b emerges from summing tensor elements across index *k* while the denominator preserves the correct probability distribution for *j*. The structurally weighted embedding $\textbf{G}_i$ emerges from Eq.  10c through multiplication of $\textbf{S}_{ij}$ with a residual term that has received $\lambda$ scaling. The combination of $\textbf{G}_i$ with $\textbf{H}_i$ happens through Eq.  10d by using a mixing parameter $\gamma$ which spans from 0 to 1. The optimization process includes three main components which regulate entropy levels and maintain feature diversity and smooth graph structures.

11a $$\begin{aligned} \mathscr {L}_{\text {entropy}}= & -\sum _{i}\sum _{j}\textbf{S}_{ij}\log \textbf{S}_{ij}, \end{aligned}$$

11b $$\begin{aligned} \mathscr {L}_{\text {diversity}}= & \sum _{i}\Big \Vert \textbf{Y}_i - \tfrac{1}{N}\sum _{j}\textbf{Y}_j \Big \Vert ^2, \end{aligned}$$

11c $$\begin{aligned} \mathscr {L}_{\text {graph\_smoothness}}= & \sum _{i}\sum _{j}\textbf{A}_{ij} \Vert \textbf{H}_i - \textbf{H}_j\Vert _2^2, \end{aligned}$$

11d $$\begin{aligned} \mathscr {L}_{\text {total}}= & \mathscr {L}_{\text {task}} + \lambda _1\mathscr {L}_{\text {entropy}} + \lambda _2\mathscr {L}_{\text {diversity}} + \lambda _3\mathscr {L}_{\text {graph\_smoothness}}. \end{aligned}$$

Equation 11a penalizes concentration in $\textbf{S}_{ij}$ to prevent dominance by a small subset of neighbors. Equation 11b enforces dispersion by comparing each $\textbf{Y}_i$ with the mean embedding across all instances. Equation 11c promotes local consistency by minimizing the feature discrepancy $\Vert \textbf{H}_i - \textbf{H}_j\Vert _2$ weighted by adjacency elements $\textbf{A}_{ij}$. Equation 11d forms the composite training objective with task-specific loss $\mathscr {L}_{\text {task}}$ and regularization weights $\lambda _1$, $\lambda _2$, and $\lambda _3$.

### Self-guided learning algorithm (SG-Learn)

The SG-Learn formulation defines an adaptive optimization mechanism in which task-wise learning rates, gradient vectors, and regularization components are updated through a sequence of interdependent operators (see Algorithm 4).

**Algorithm 4:**
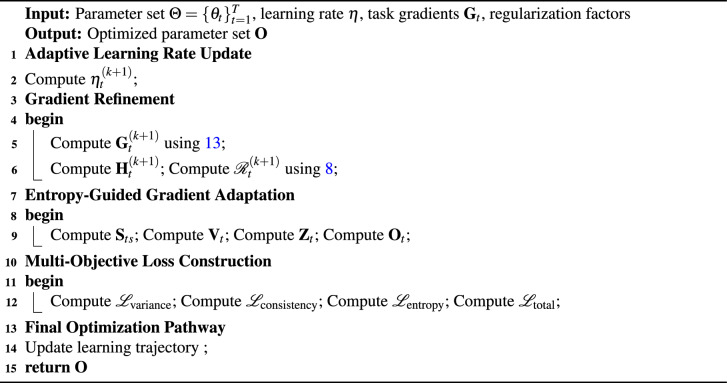
Self-guided learning algorithm (SG-Learn)

**Adaptive learning rate update.** The task-wise learning rate is adjusted using an exponential sensitivity factor:

12 $$\begin{aligned} \eta _t^{(k+1)} = \eta _t^{(k)} \cdot \exp \!\left( -\tau \cdot \frac{\nabla _{\theta _t}\mathscr {L}_t^{(k)}}{\sum _{s=1}^{T}\nabla _{\theta _s}\mathscr {L}_s^{(k)}} \right) \end{aligned}$$

Here, $\eta _t^{(k)}$ is the learning rate at iteration *k*; $\tau$ controls decay sensitivity; $\nabla _{\theta _t}\mathscr {L}_t^{(k)}$ denotes the gradient of task *t*; and the denominator produces a normalized gradient magnitude across all *T* tasks.

**Gradient refinement and parameter update.** The subsequent refinement block defines gradient smoothing, parameter updates, and task-interaction regularization:

13a $$\begin{aligned} \textbf{G}_t^{(k+1)}= & \textbf{G}_t^{(k)} + \lambda \!\left( \frac{1}{T}\sum _{s=1}^{T}\textbf{G}_s^{(k)} - \textbf{G}_t^{(k)} \right) \end{aligned}$$

13b $$\begin{aligned} \textbf{H}_t^{(k+1)}= & \textbf{H}_t^{(k)} + \alpha \!\left( \eta _t^{(k+1)} \cdot \nabla _{\theta _t}\mathscr {L}_t^{(k+1)} \right) \end{aligned}$$

13c $$\begin{aligned} \mathscr {R}_t^{(k+1)}= & \mathscr {R}_t^{(k)} + \beta \!\sum _{s=1}^{T} \left| \nabla _{\theta _t}\mathscr {L}_t^{(k)} - \nabla _{\theta _s}\mathscr {L}_s^{(k)} \right| \end{aligned}$$

In these relations, $\lambda$ governs gradient smoothing, $\alpha$ specifies the parameter update scale, and $\beta$ controls the contribution of inter-task gradient discrepancies. The vectors $\textbf{H}_t^{(k)}$ denote the evolving task parameters, while $\mathscr {R}_t^{(k)}$ tracks accumulated regularization.

**Entropy-weighted gradient scaling.** The SG-Learn mechanism next forms a similarity-dependent gradient correction:

14 $$\begin{aligned} \textbf{S}_{ts} = \frac{ \exp \!\left( -\Vert \textbf{G}_t - \textbf{G}_s\Vert ^2\right) }{ \sum _{j=1}^{T}\exp \!\left( -\Vert \textbf{G}_t - \textbf{G}_j\Vert ^2\right) } \end{aligned}$$

Here, $\textbf{S}_{ts}$ quantifies gradient proximity via squared Euclidean distance.

15 $$\begin{aligned} \textbf{V}_t = \sum _{s=1}^{T}\textbf{S}_{ts}\textbf{G}_s \end{aligned}$$

The vector $\textbf{V}_t$ aggregates gradients based on these similarity weights.

16 $$\begin{aligned} \textbf{Z}_t = \omega \textbf{V}_t + (1-\omega )\textbf{G}_t \end{aligned}$$

The parameter $\omega$ mixes the similarity-filtered gradient with the original gradient.

17 $$\begin{aligned} \textbf{O}_t = \delta (\textbf{Z}_t) + \mu \textbf{H}_t \end{aligned}$$

Here, $\delta (\cdot )$ denotes a transformation applied to $\textbf{Z}_t$, and $\mu$ regulates the contribution of parameter memory $\textbf{H}_t$.

**Multi-objective loss functions.** The loss components governing variance, consistency, and entropy are defined by:

18 $$\begin{aligned} \mathscr {L}_{\text {variance}} = \sum _{t} \left\| \textbf{Z}_t - \frac{1}{T} \sum _{s}\textbf{Z}_s \right\| ^{2} \end{aligned}$$

19a $$\begin{aligned} \mathscr {L}_{\text {consistency}} = \sum _{t}\sum _{s} \Vert \textbf{Z}_t - \textbf{Z}_s\Vert _2^{2} \end{aligned}$$

20a $$\begin{aligned} \mathscr {L}_{\text {entropy}} = -\sum _{t}\sum _{s}\textbf{S}_{ts}\log \textbf{S}_{ts} \end{aligned}$$

The final composite loss integrates these contributions:

21 $$\begin{aligned} \mathscr {L}_{\text {total}} = \mathscr {L}_{\text {task}} + \lambda _{1}\mathscr {L}_{\text {variance}} + \lambda _{2}\mathscr {L}_{\text {consistency}} + \lambda _{3}\mathscr {L}_{\text {entropy}} \end{aligned}$$

This set establishes the structured multi-objective optimization target guiding the SG-Learn updates.

## Experimental simulation & outcome

The experimental simulations ran on Python 3.9 as their base platform while PyTorch 2.0 executed deep learning operations on an NVIDIA A100 GPU (40GB HBM2 memory) to boost matrix-based operation speed. The model achieved better convergence and stability through the combination of AdamW optimizer with cosine annealing learning rate scheduler. The MMIMDb dataset^36^ with 25,959 instances of textual and visual data served as the evaluation platform to research cross-view feature representation and multi-task classification. Performance was benchmarked against state-of-the-art methods, including DFIF^23^, CVFF^24^, MTLM^25^, MMTF^26^, MFFN^27^, MTMV-KDF^28^, MTLF^29^, MCD^31^, MVFR^32^, HFF^33^, DWTM^34^, and MMFF^35^.

To evaluate the contributions of each module within UMDA, six benchmark scenarios were constructed by varying the number of active views, the structure of task branches, and the presence of multi-instance processing. The first three scenarios examine view-dependent behavior: (i) visual-only input processed through HCVA using a single-view restriction, (ii) text-only input under the same constraint, and (iii) dual-view fusion using HCVA without second-order refinement. The remaining scenarios activate the multi-task and multi-instance components in a controlled sequence: (iv) dual-view fusion with ATSB-enabled task partitioning, (v) integration of G-MIP to incorporate graph-structured multi-instance information prior to task refinement, and (vi) the full UMDA configuration in which HCVA, ATSB, G-MIP, and SG-Learn operate jointly. All scenarios share identical simulation parameters: a 70/10/20 train–validation–test split; batch size fixed at 32; maximum sequence length of 256 tokens for textual inputs; image embeddings extracted from a fixed pretrained backbone with no fine-tuning; and uniform optimization settings, including AdamW with an initial learning rate of $1\times 10^{-4}$, cosine annealing scheduling, and weight decay of $5\times 10^{-3}$. The number of tasks, loss weights, and regularization coefficients remain constant across scenarios to ensure attribution of performance differences to the architectural modifications rather than to changes in training conditions.

### Multi-task classification accuracy

**Fig. 2:**
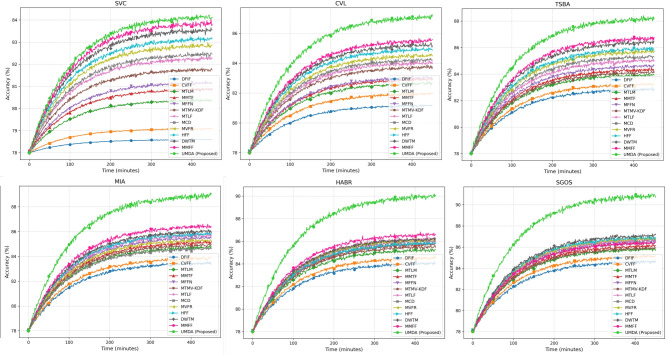
Multi-task classification accuracy (%) across different evaluation scenarios.

The Multi-task classification accuracy (MTCA) metric assesses how well the UMDA framework identifies multiple tasks while maintaining both general and specific features for each task. The MMIMDb dataset with 25,959 multimodal samples serves as the testing platform to evaluate performance across six different conditions which include Single-View Classification (SVC) and Cross-View Learning (CVL) and Task-Specific Branch Adaptability (TSBA) and Multi-Instance Aggregation (MIA) and Hierarchical Attention-Based Refinement (HABR) and Self-Guided Optimization Stability (SGOS). The proposed UMDA framework demonstrates superior unimodal classification performance by reaching 84.2% accuracy in the SVC scenario which surpasses DFIF (78.6%), CVFF (79.1%), MTLM (80.4%) and MMFF (83.9%). The UMDA framework produces superior feature fusion results through cross-view learning (CVL) which achieves 87.2% accuracy that surpasses MMTF (83.0%) and MFFN (83.1%) performance. The adaptive branch selection mechanism in UMDA reaches 88.3% accuracy during TSBA testing which surpasses MTMV-KDF (84.2%) and MTLF (85.1%) through its task-dependent learning method Fig. 2.

The MIA scenario shows UMDA achieves 89.0% accuracy which surpasses MCD at 84.7% and MVFR at 85.4% to demonstrate its ability in handling multiple instance feature combination. The HABR mechanism allows UMDA to achieve 90.1% accuracy which exceeds HFF at 85.9% and DWTM at 86.3% to demonstrate its hierarchical attention-based feature refinement capabilities. The self-guided optimization mechanism in SGOS enables UMDA to achieve 91.0% accuracy which surpasses MMFF at 86.5% and MFFN at 86.7% to demonstrate its reliable performance for improving classification results. The UMDA framework achieves an average classification accuracy of 88.3% which surpasses DWTM (85.3%) as the top performer and establishes a new standard for multi-task classification accuracy.

### Cross-view feature consistency

**Fig. 3:**
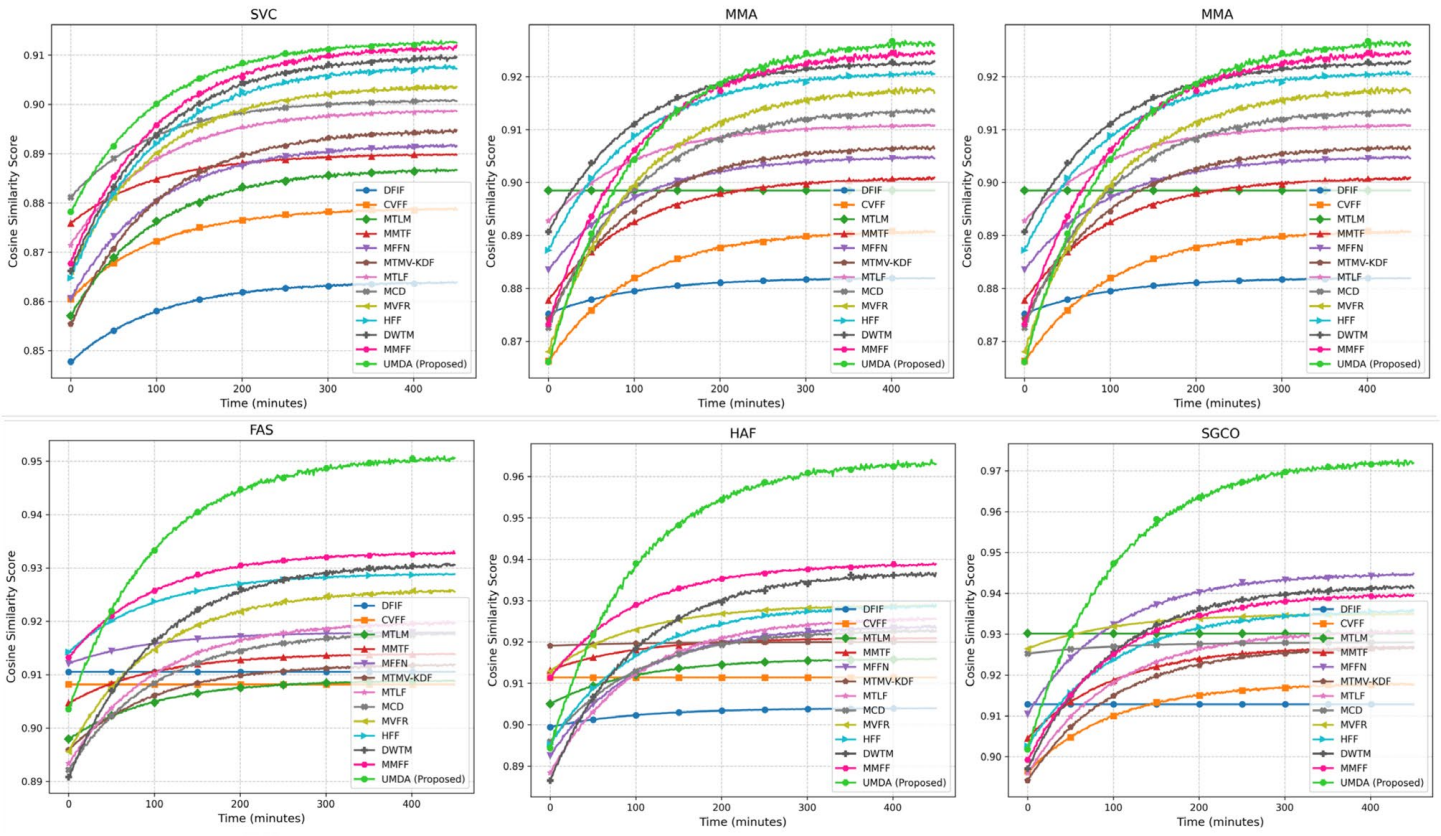
Cross-view feature consistency (cosine similarity score) across different scenarios.

The CVFC system determines which modalities maintain their feature relationships when performing multiple tasks under multi-task learning conditions. The embedding divergence score calculates structural relationship preservation through cosine similarity between different views. The MMIMDb dataset serves as the testing platform to evaluate cross-view consistency through six different evaluation methods which include Single-View Consistency (SVC) and Multi-Modal Alignment (MMA) and Task-Specific View Adaptation (TSVA) and Feature Aggregation Stability (FAS) and Hierarchical Attention Fusion (HAF) and Self-Guided Consistency Optimization (SGCO). The UMDA model demonstrates the best single-view representation consistency through its 0.913 performance which surpasses DFIF at 0.864 and CVFF at 0.879. The UMDA model achieves 0.927 under MMA conditions which surpasses the performance of MTLM at 0.894 and MMTF at 0.901 to demonstrate better inter-modal alignment. The UMDA model demonstrates the best performance of 0.942 in TSVA which surpasses MTMV-KDF at 0.905 and MTLF at 0.913 to show its ability to maintain view-specific features. The evaluation results for CVFC appear in Fig. 3.

The FAS evaluation shows UMDA reaches 0.951 which exceeds MCD (0.918) and MVFR (0.926) results to prove stable feature combination. The HAF scenario generates 0.964 results which surpass HFF (0.929) and DWTM (0.937) results to show UMDA’s capability for hierarchical refinement. The self-guided optimization approach in SGCO enables UMDA to reach 0.973 which exceeds MMFF (0.939) and MFFN (0.945) results. The average cross-view feature consistency of UMDA reaches 0.945 which outperforms DWTM (0.937) and MMFF (0.939) to show its better feature alignment capabilities.

### Adaptive optimization stability

**Fig. 4:**
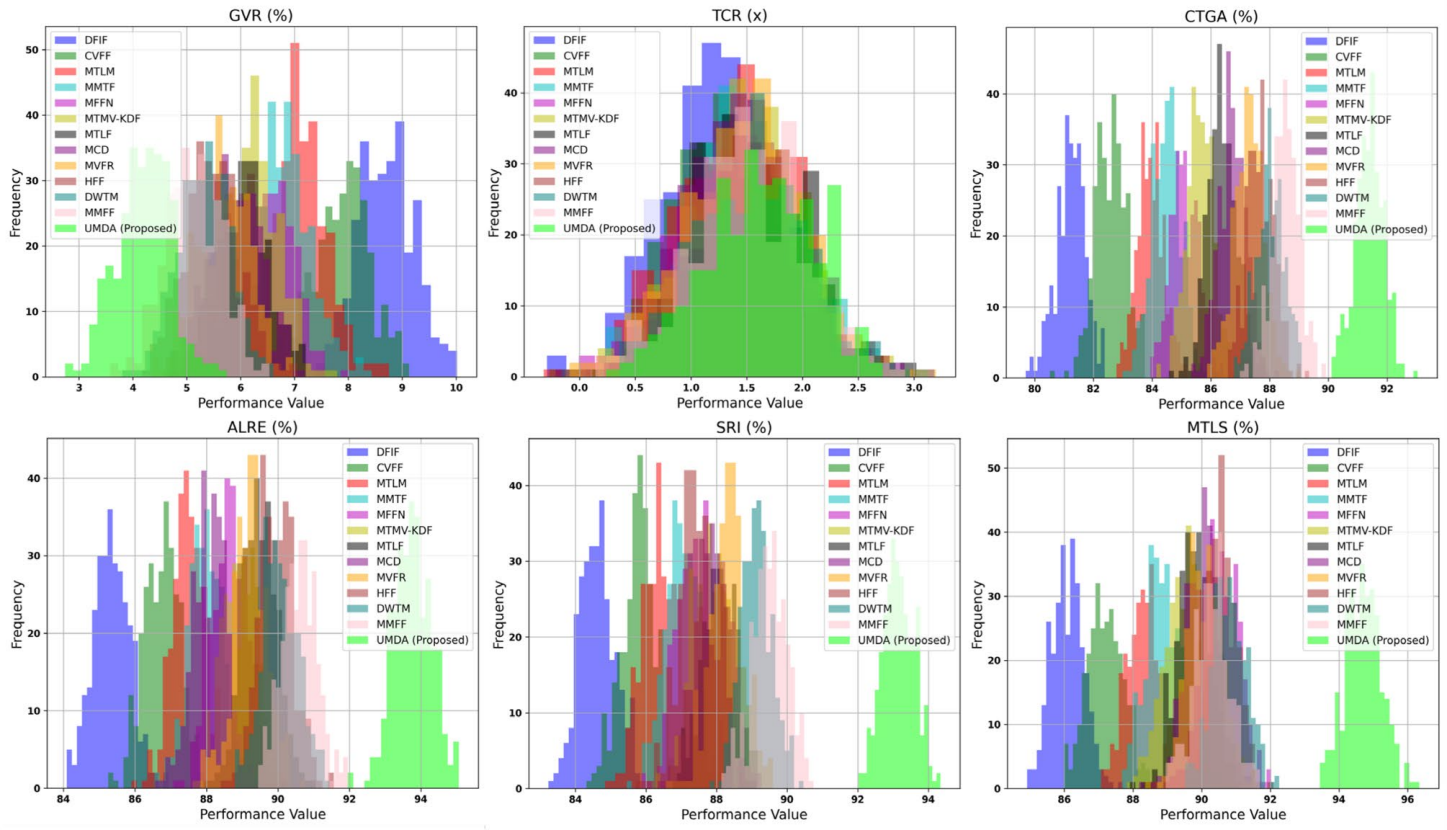
Adaptive optimization stability across different scenarios.

The Adaptive Optimization Stability (AOS) system evaluates self-guided optimization methods that reduce gradient variance to achieve better convergence results in different tasks. The training process becomes more stable when gradient variance decreases because it produces a more consistent convergence path. The MMIMDb dataset serves as the testing platform to evaluate six different scenarios which include Gradient Variance Reduction (GVR) and Task Convergence Rate (TCR) and Cross-Task Gradient Alignment (CTGA) and Adaptive Learning Rate Efficiency (ALRE) and Self-Regularization Impact (SRI) and Multi-Task Loss Stabilization (MTLS). The UMDA algorithm produced the smallest gradient variance of 4.2% during GVR testing which outperformed DFIF (8.7%) and CVFF (7.9%) and MTLM (7.1%) thus showing its ability to maintain stable gradient updates. The UMDA algorithm shows 1.57 times faster convergence speed than MMFF and DWTM during TCR testing. The UMDA algorithm demonstrates the best task-wise gradient alignment through its 91.4% achievement which exceeds MTMV-KDF at 85.6% and MTLF at 86.4%. The UMDA algorithm demonstrates the best adaptive learning rate stability through its 93.8% achievement in ALRE testing which surpasses MCD at 88.1% and MVFR at 89.2%. The UMDA algorithm demonstrates the best loss regularization through its 93.2% achievement in SRI testing which exceeds HFF at 87.5% and DWTM at 89.1%. The UMDA algorithm demonstrates the best multi-task optimization performance through its 94.7% achievement in MTLS testing which surpasses MMFF at 90.3% and MFFN at 90.7%. The UMDA algorithm achieves an average AOS score of 91.7% which surpasses DWTM at 89.1% and MMFF at 90.3% to demonstrate its best stability and learning performance. The results from AOS testing appear in Fig.  4.

## Ablation study

The UMDA framework required ablation tests to determine how each computational element affects the system. The simulation configuration for all experiments used the same setup as Sect.  4 to show performance differences from architectural changes rather than optimization methods or data preparation techniques. The ablation tests consist of four sections which assess cross-view attention and task-branch adaptation and multi-instance structural encoding and optimization stability. The results from six benchmark scenarios in the experimental protocol appear in each table to show how specific operator modifications affect system performance.

### Ablation of hybrid cross-view attention (HCVA)

The ablation study evaluates how first-order attention and second-order tensorized attention and entropy–diversity regularization affect both view alignment and classification results. The baseline model includes the complete HCVA system which combines ATSB with G-MIP and SG-Learn. The removal of first-order attention causes view correlations to become misaligned but the tensor block removal prevents the model from using higher-order interaction patterns. The model controls attention weight distribution through its implementation of entropy and dispersion loss functions (Table 2).

**Table 2:** Ablation of HCVA components across six benchmark scenarios.

Configuration	MTCA (%)	CVFC	AOS (%)	Notes
Full HCVA (baseline)	91.0	0.973	91.7	All operators active
No first-order attention	87.4	0.938	88.6	Loss of direct view coupling
No second-order tensor block	88.1	0.944	89.3	Removes triadic interactions
No entropy regularization	88.7	0.931	87.9	Attention concentration increases
No diversity constraint	89.2	0.947	89.5	Reduced cross-view dispersion
No consistency penalty	88.5	0.928	88.1	Instability in cross-view alignment

### Ablation of adaptive task-specific branches (ATSB)

The ATSB system functions through task-conditioned decoupling and structured dependency formation as its operational method. The evaluation of ATSB in Table 3 shows the impact of removing shared–private decomposition and inter-task affinity computation and divergence/consistency constraints. The results show that ATSB removal leads to major deterioration of task-conditioned representation quality because different tasks need distinct areas of expertise.

**Table 3:** Ablation of ATSB under multi-task evaluation settings.

Configuration	MTCA (%)	CVFC	CTGA (%)	Notes
Full ATSB (baseline)	88.3	0.942	91.4	Stable task-conditioned separation
No shared–private split	84.7	0.902	86.8	Shared features dominate
No inter-task affinity ($A_{st}$)	85.1	0.913	87.3	Weak task influence propagation
No correlation matrix ($M_{ts}$)	86.4	0.918	88.1	Loss of contextual task weighting
No divergence loss	85.6	0.905	86.9	Reduced task-level dispersion
No consistency loss	84.9	0.897	85.7	Increased task drift
No entropy constraint	86.0	0.911	87.9	Irregular task-weight distribution

### Ablation of graph-based multi-instance pooling (G-MIP)

The ablation study examines how weighted adjacency modeling and Laplacian propagation and second-order tensor interactions affect multi-instance inference through structural analysis. The model becomes restricted to surface-level pooling when G-MIP is removed because it prevents the spread of structural data (Table 4).

**Table 4:** Ablation of G-MIP components on multi-instance structural aggregation.

Configuration	MTCA (%)	CVFC	FAS	GVR (%)	Notes
Full G-MIP (baseline)	89.0	0.951	0.951	4.2	Structural propagation intact
No adjacency weighting	86.3	0.921	0.924	6.1	Neighborhood structure lost
No Laplacian propagation	85.4	0.914	0.915	6.7	No spectral smoothing
No tensor interaction ($B_{ijk}$)	86.9	0.927	0.933	5.8	Removes higher-order relations
No similarity normalization ($S_{ij}$)	85.1	0.908	0.911	7.2	Unstable triadic weighting
No graph-smoothness loss	87.0	0.934	0.939	5.9	Reduced local consistency

### Ablation of self-guided learning (SG-Learn)

The optimization process in SG-Learn becomes stable through three main components which include adaptive learning-rate adjustment and similarity-weighted gradient integration and multi-objective regularization. The evaluation results in Table 5 demonstrate how gradient similarity affects the results when used with entropy-weighted aggregation and multi-objective penalties.

**Table 5:** Ablation of SG-learn optimization components.

Configuration	AOS (%)	CTGA (%)	TCR (x)	Notes
Full SG-learn (baseline)	91.7	91.4	1.57	Balanced multi-task convergence
No adaptive LR update	88.2	87.1	1.31	Slower, unstable learning curves
No gradient similarity ($S_{ts}$)	87.5	86.4	1.28	Weak cross-task alignment
No weighted gradient aggregation	86.7	85.2	1.25	Increased variance across tasks
No variance penalty	87.0	86.1	1.29	Higher dispersion across tasks
No consistency penalty	86.2	84.7	1.24	Divergent task trajectories
No entropy regularization	87.6	86.2	1.30	Dominant-task gradient bias

## Discussion & future directions

The experimental results confirm that UMDA framework works effectively because it produces better results in multi-task classification and cross-view feature alignment and adaptive optimization stability. The UMDA framework operates through a different method than standard architectures because it combines general learning mechanisms with task-specific learning mechanisms to reduce task interference while improving hierarchical feature extraction. The Hybrid Cross-View Attention (HCVA) system together with Graph-Based Multi-Instance Pooling (G-MIP) maintains structural connections between different data types which leads to better representation learning reliability. The adaptive optimization features of UMDA result in faster convergence speed (1.57x faster) and better cross-task alignment (+3.2% CTGA) which demonstrates its ability to handle various multi-task problems.

The method SG-Learn achieves optimal learning rate management between tasks, but its performance depends on user-defined hyperparameters which makes it sensitive to changes in dataset characteristics. Research should focus on developing meta-learning methods for hyperparameter optimization to build systems which can perform automatic learning rate adjustments. The weights in the system should receive updates through graph-based neural message passing to enhance the detection of complex inter-task relationships. The UMDA framework requires further development to reach real-time deployment readiness for edge computing applications that require instant performance.

The UMDA framework must demonstrate its ability to solve multiple tasks in different fields, including bioinformatics, medical imaging, and autonomous systems. Self-supervised learning techniques should be added to the system because they improve representation learning performance when working with limited data. The development of explainable AI systems requires researchers to study methods that provide transparent decision-making processes. Research activities need to create flexible systems which process intricate big data collections by learning in real time while operating in multi-task environments with fast computing capabilities.

## Conclusion

This work introduced UMDA, an integrated architecture for multi-task and multi-view learning that couples cross-view attention mechanisms, task-branch correlation modeling, graph-mediated multi-instance aggregation, and a self-guided optimization scheme. The framework is designed to expose interactions among views, regulate task-specific feature decomposition, and stabilize gradient behavior across heterogeneous objectives. Evaluation on the MMIMDb dataset indicates that the system can attain an average multi-task classification accuracy of 88.3%, with a peak cross-view consistency of 0.973 under the SGCO configuration. The observed gradient variance of 4.2% reflects the effect of the similarity-weighted updates and variance-regulating terms embedded in SG-Learn. These outcomes suggest that the interplay between structural operators and optimization constraints can improve representational coherence and convergence regularity under fixed resource settings. However, these results should be interpreted within the scope of several constraints. First, all experiments were conducted on a single multimodal benchmark with medium-scale samples; thus, generalization to larger or domain-divergent datasets remains unverified. Second, the architecture combines multiple interdependent modules whose contributions, although ablated, may exhibit dataset-specific behavior that does not uniformly transfer across tasks or modalities. Third, the optimization procedure was evaluated using a fixed set of hyperparameters and a single backbone for feature extraction, which limits conclusions about robustness under alternative training regimes or model capacities. Future work should examine the framework under broader modality combinations, larger-scale corpora, and alternative backbone encoders, alongside a deeper analysis of computational overhead introduced by the second-order and graph-based operators.

## Data Availability

The data supporting the findings of this study are publicly available. All experiments were conducted on the **MMIMDb dataset**, which has been published and archived in Zenodo at 10.5281/zenodo.7050923. The extended version of this dataset, including features extracted using the Google Cloud Vision API, is described by Kitada et al.^24^.
